# Circulating Monocyte Subsets Are Associated With Extent of Myocardial Injury but Not With Type of Myocardial Infarction

**DOI:** 10.3389/fcvm.2021.741890

**Published:** 2021-11-02

**Authors:** Noushin Askari, Christoph Lipps, Sandra Voss, Nora Staubach, Dimitri Grün, Roland Klingenberg, Beatrice von Jeinsen, Jan Sebastian Wolter, Steffen Kriechbaum, Oliver Dörr, Holger Nef, Christoph Liebetrau, Christian W. Hamm, Till Keller

**Affiliations:** ^1^Department of Internal Medicine I, Cardiology, Justus-Liebig-University Gießen, Giessen, Germany; ^2^Department of Cardiology, Kerckhoff Heart and Thorax Center, Bad Nauheim, Germany; ^3^German Center for Cardiovascular Research e.V. (DZHK), Partner Site RhineMain, Bad Nauheim, Germany; ^4^Cardiovascular Center Bethanien (CCB), Frankfurt, Germany

**Keywords:** monocytes, acute coronary syndrome, inflammation, biomarker, troponin, chronic coronary syndrome

## Abstract

Inflammation is a hallmark of the period after a myocardial infarction (MI) that is either promoted or resolved by distinct subtypes of circulating inflammatory cells. The three main monocyte subpopulations play different roles inflammation. This study examined whether the type of MI (type 1 or type 2) or the extent of myocardial injury is associated with differences in monocyte subpopulations. For this purpose, peripheral whole blood from patients with a suspected MI was used for flow cytometric measurements of the monocyte subpopulations, and myocardial injury was classified by cardiac troponin levels in serum. In patients with acute coronary syndrome (*n* = 82, 62.2% male) similar proportions of the monocyte subsets were associated with the two types of MI, whereas total monocyte counts were increased in patients with substantial myocardial injury vs. those with minor injury (*p* = 0.045). This was accompanied by a higher proportion of intermediate (*p* = 0.045) and classical monocytes (*p* = 0.059); no difference was found for non-classical monocytes (*p* = 0.772). In patients with chronic coronary syndrome (*n* = 144, 66.5% male), an independent association with myocardial injury was also observed for classical monocytes (*p* = 0.01) and intermediate monocytes (*p* = 0.08). In conclusion, changes in monocyte subpopulation counts, particularly for classical and intermediate monocytes, were related to the extent of myocardial injury in acute and stable coronary artery disease but not to the type of MI.

## Introduction

Myocardial infarction (MI) is one of the most frequent cardiovascular events and leads to relevant morbidity and mortality. Early inflammatory processes after MI are largely dependent on monocyte-related mechanisms (1, 2). It has been established that following the onset of acute MI, monocytes invade the infarcted area to promote healing of damaged myocardial tissue by removing dead cells and have an important impact on remodeling of myocardial structure (2).

Monocytes represent ~5% of peripheral blood leucocytes; they constitute an essential component of the immune system linking innate and adaptive immunity and are critical drivers in many inflammatory processes (3). This is also the case in atherosclerosis, in which monocytosis after an MI is associated with impaired recovery and unfavorable prognosis of atherosclerotic disease (4, 5).

Three different monocyte subsets can be distinguished, as delineated by the Nomenclature Committee of the International Union of Immunological Societies in 2010. Thus, monocytes are divided into classical monocytes (CD14++CD16–), representing up to 90% of the blood monocytes, intermediate monocytes (CD14++CD16+), and non-classical monocytes (CD14+CD16++) (6). These three monocyte subsets have distinct phenotypic and functional characteristics and play different roles in inflammation and malignancy. Each subset displays different immune functions, including phagocytic activity, cytokine profile, and regenerative capacity (7). Classical monocytes are characterized by a high expression of genes encoding antimicrobial proteins (7). Non-classical cells display numerous patrolling properties and therefore are thought to be involved in innate surveillance of tissues (7). Intermediate monocytes constitute a transitional population that shares some phenotypic and functional features of both classical and non-classical monocytes (7). It has been suggested that the proportion of the three subpopulations of monocytes can differ with the presence of disease, such as the different characteristics associated with different types of MI (8).

MI can be classified by the universal definition as type 1, caused by a spontaneous coronary plaque rupture, and type 2, resulting from increased oxygen demand or decreased supply (9). MI in general is characterized by cell death due to prolonged ischemia, irrespective of the type of MI. Myocardial cell death as related to the extent of myocardial injury can be quantified by measuring different muscle-associated proteins released into the circulation from the damaged myocytes, including cardiac troponins, that serve as biomarkers with high myocardial specificity (10).

The aim of our study was to evaluate whether the type of MI and the extent of myocardial injury are associated with the inflammatory profile and quantity of circulating monocyte subpopulations.

## Materials and Methods

### Clinical Cohorts

The present analyses are based on two independent multicenter cohorts located at the Kerckhoff Heart Center in Bad Nauheim, Germany, and the University Hospital of the Justus-Liebig-University in Giessen, Germany. The two studies were approved by the local Ethics Committee of the University Giessen (AZ 199/15) and each patient gave written informed consent. The studies were carried out in accordance with the Declaration of Helsinki.

The first cohort, referred to as the acute coronary syndrome (ACS) cohort, utilized data and biomaterial from a prospective, multicenter biomarker registry that enrolled patients presenting with suspected acute MI between August 2011 and October 2016, as described previously (11). Here, the final gold-standard study diagnosis, including type of MI, was made independently by two cardiologists based on the Third Universal Definition of Myocardial Infarction (9) using all available clinical, laboratory, and imaging data. In this ACS cohort, flow cytometry data and troponin I values were available in 101 patients.

For the second cohort, referred to as the chronic coronary syndrome (CCS) cohort, data and biomaterial were used from an ongoing multicenter biomarker registry that enrolled patients with suspected CCS starting in August 2010, as described previously (12). This cohort comprised patients with a clinical indication for invasive coronary angiography due to suspected CCS, including patients with and without previously known coronary artery disease. In this CCS cohort, flow cytometry data and troponin I values were available in 144 patients.

### Laboratory Analyses and Definition of Myocardial Injury

Standard laboratory parameters such as cholesterol or creatinine levels were measured directly upon enrollment via the respective central laboratories of the recruiting centers. At study enrollment blood samples were taken, centrifuged, aliquoted and stored at −80°C according to standard operating procedures in both cohorts.

To define myocardial injury (13), troponin I in serum samples was measured batchwise with two commercially available troponin I assays. High-sensitive cardiac troponin I (hs-cTnI) was measured in the ACS cohort using an automated immunoassay (ARCHITECT STAT high-sensitive troponin, Abbott Diagnostics, Abbott Park, IL, USA) with a limit of detection (LOD) of 1.9 ng/L, a limit of quantitation (LOQ) of 10 ng/L, and a 99th percentile concentration of 26.2 ng/L (used to define relevant myocardial injury) according to the manufacturer's specifications. To take into account the potentially smaller amount of myocardial injury in patients without MI, a super-sensitive cardiac troponin I (ss-cTnI) was measured in the CCS cohort using a single-molecule array (Simoa Troponin-I, Quanterix, Billerica, MA, USA) on the Quanterix SR-X system with an LOD of 0.021 pg/mL and an LOQ of 0.122 pg/mL according to the manufacturer's information. As no established threshold is available in the context of CCS, we used the median concentration of 3.0 ng/mL to signify the presence of myocardial injury. In both ACS and CCS cohorts, patients were grouped according to the troponin I levels: those in the “minor” injury group had troponin levels below the respective threshold and those in the “substantial” injury group had levels that were higher.

### Flow Cytometry

To determine absolute counts of monocyte subsets, 50 μl of freshly collected EDTA-anticoagulated whole blood were transferred into TruCount Absolute Counting Tubes (BD Biosciences, San Jose, CA, USA) by reverse pipetting and stained immediately with fluorophore-labeled monoclonal antibodies directed against the monocyte key markers CD14 (Clone M5E2), CD16 (Clone 3G8), and CD11b (Clone ICRF44) (BioLegend, San Diego, CA, USA) as well as a granulocyte marker (CD66b, Clone G10F5, BioLegend). Erythrocytes were lysed using Pharm Lyse Buffer (BD Biosciences) before acquiring specimen data on a FACSVerse flow cytometer (BD Biosciences). Instrument performance was tracked daily with BD FACSuite CS&T Beads (BD Biosciences), and assay-specific settings were updated daily to ensure proper assay reproducibility.

The resulting FCS files were exported in FCS 3.0 format and analyzed using FACSDiva software (Version 6.3.1, BD Biosciences). An exemplary dataset taken from the ACS cohort is shown in Supplementary Figure 1. Absolute counting beads were identified as highly fluorescent events with low forward scatter. All events outside the bead region were displayed in a two-dimensional dot plot to exclude events with low forward scatter and low side scatter from the analysis. Granulocytes, defined as events with high side scatter and high CD66b staining intensity, were excluded in the next step. Monocytes were identified in the remaining events as a population with intermediate side scatter and high CD11b expression, excluding lymphocytes from the analysis. Events in the monocyte gate were further divided into the three monocyte subsets according to their CD14 and CD16 staining intensity: CD14++ CD16- classical monocytes, CD14++ CD16+ intermediate monocytes, and CD14+ CD16++ non-classical monocytes. Cell counts were calculated according to the manufacturer's protocol.

### Statistical Analyses

Data are presented as mean with standard deviation (SD) for normally distributed variables and as median with interquartile range (IQR) for skewed continuous variables. For dichotomous variables data are given as absolute number and percentage. Fischer's exact test, Student's *t*-test, or the Mann-Whitney *U*-test were applied, as appropriate, to test for differences between groups. Spearman correlations were calculated between monocytes subsets and peak creatine kinase (CK) values as marker for peak myocardial injury during the individual ACS time course. Further, the potential independent associations of troponin as marker for myocardial injury with monocyte subsets were checked in the ACS and the CCS cohort by using multivariate regression analysis. Not normally distributed variables were logarithmic transformed in the evaluation. All calculated and presented *P*-values should be viewed as descriptive. All statistical analyses were carried out using the R 3.6.1 software package (R Foundation for Statistical Computing, Vienna, Austria).

## Results

### Type of MI and Extent of Myocardial Injury in the Acute Coronary Syndrome Cohort

The ACS cohort comprised 101 patients with suspected acute MI. Of those, 62 patients (69% male, age 59–78 years) experienced an MI, whereas in 39 patients (59% male, age 59–76 years) an MI could be excluded. MI patients were further classified according to the final diagnosis of MI type: MI type 1, the prototypical acute MI (T1MI, *n* = 52, 73% male, age: 59–78 years) or MI type 2, an MI based on ischemia due to an oxygen demand/supply mismatch (T2MI, *n* = 10, 50% male, age: 61–81 years). T1MI and T2MI patients did not differ regarding their cardiovascular risk profile. The baseline characteristics of the ACS cohort stratified by MI type are provided in Supplementary Table 1.

To evaluate the influence of the extent of myocardial damage irrespective of the final diagnosis given, patients of the entire ACS cohort were classified as having minor or substantial myocardial injury based on the troponin I level. Troponin I data were available in 82 patients. In 42 patients substantial myocardial damage was present (61.9% male, age: 58–81 years), whereas 40 patients showed only minor myocardial injury (62.5% male, age: 64–75 years). In the group of patients with minor myocardial damage, a higher proportion (55.9%) tended to have previously known coronary artery disease compared to patients with substantial injury (44%, *p* = 0.066). Baseline characteristics of the ACS cohort stratified according to extent of myocardial injury are provided in Table 1.

**Table 1:** Baseline characteristics of the acute coronary syndrome (ACS) cohort.

**Acute coronary syndrome (ACS) cohort**	**Type and unit**	**Overall ACS cohort**	**Substantial myocardial injury**	**Minor myocardial Injury**	***p*–value**
		***n* = 82**	***n* = 42**	***n* = 40**	
Age	Median (IQR), [years]	69.52 (58.89–78.47)	71.26 (57.56–80.47)	68.61 (63.5–75.43)	0.702
Male sex	*n* (%)	51 (62.2)	26 (61.9)	25 (62.5)	1
**Final diagnosis**					
Acute myocardial infarction	*n* (%)	48 (58.54)	38 (90.48)	10 (25)	<0.001
**Cardiovascular risk factors**					
Arterial hypertension	*n* (%)	58 (79.45)	26 (76.47)	32 (82.05)	0.091
Dyslipidemia	*n* (%)	39 (55.71)	19 (55.88)	20 (55.56)	0.825
Diabetes mellitus	*n* (%)	21 (28.77)	9 (26.47)	12 (30.77)	0.452
Smoking	*n* (%)	29 (50.88)	14 (53.85)	15 (48.39)	0.818
Family history	*n* (%)	15 (28.3)	7 (26.92)	8 (29.63)	0.779
**History**					
Known coronary artery disease	*n* (%)	30 (50.85)	11 (44)	19 (55.88)	0.066
**Laboratory analyses**					
eGRF	median (IQR), [ml/min/1.73 m^2^]	86.17 (58.28–104.84)	87.13 (55.52–104.45)	83.12 (66.54–103.66)	0.612
Creatinine	median (IQR), [mg/dl]	0.87 (0.75–1.11)	0.86 (0.73–1.12)	0.87 (0.78–1.07)	0.545
hs-cTnI	median (IQR), [pg/mL]	28.2 (4–531.35)	525.6 (214.03–2,986.82)	4 (2.55–7.25)	<0.001
Cholesterol	mean ± SD, [mg/dl]	201.91 ± 45.52	211 ± 45.93	184.88 ± 42.25	0.19
C-reactive protein	median (IQR), [mg/dl]	0.3 (0.2–1.4)	0.4 (0.18–1.52)	0.3 (0.2–1)	0.82

*Data are shown stratified according to the extent of myocardial injury defined by high sensitivity cardiac troponin I values (99th percentile cut-off)*.

*Data are presented as percentage, mean, or median as appropriate. eGFR denotes estimated glomerular filtration rate, hs-cTnI denotes high sensitivity cardiac troponin I*.

### Monocyte Subpopulations in Different Types of MI

**Figure 1:**
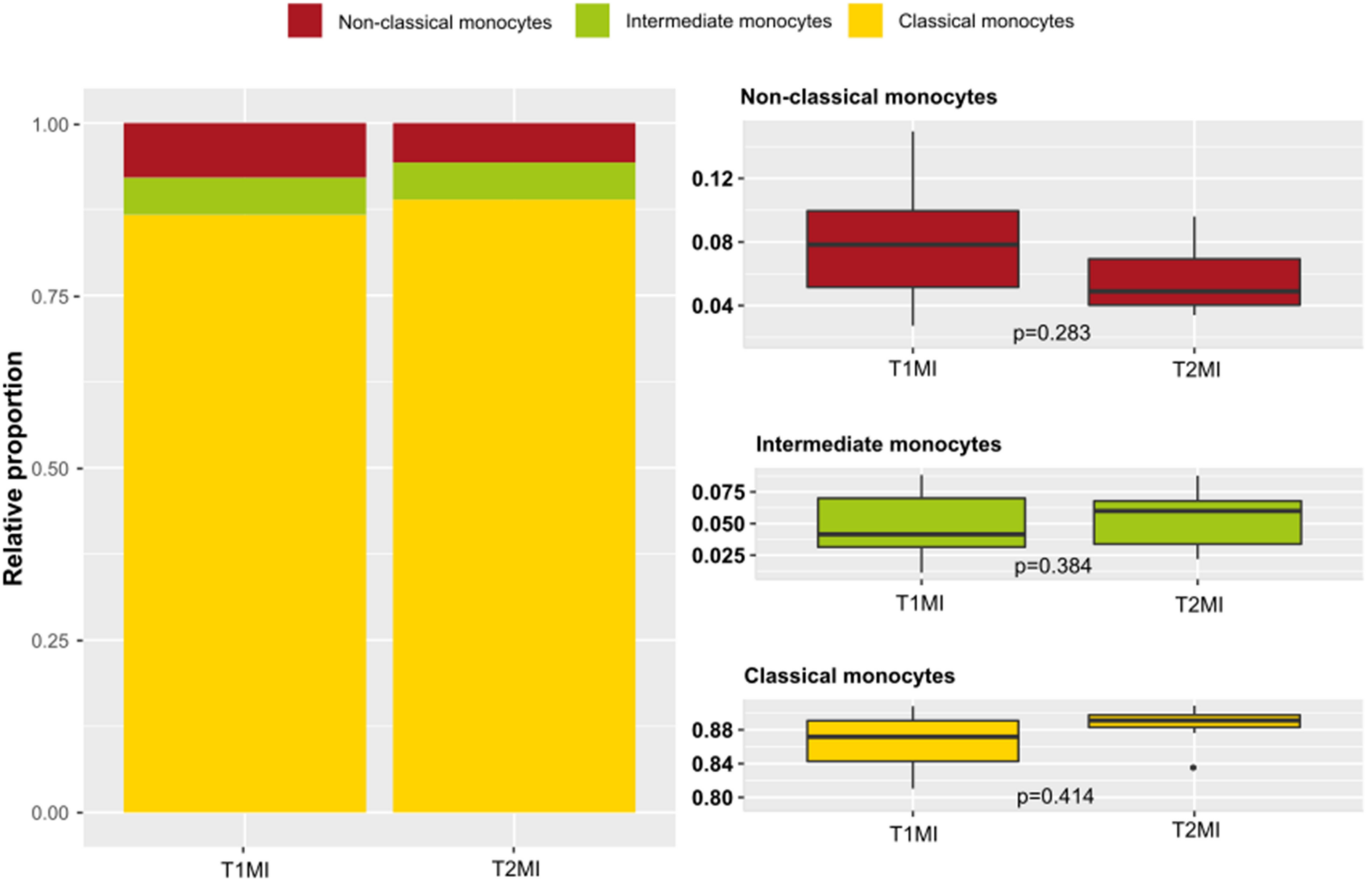
Monocyte subsets in myocardial infarction (MI) type 1 vs. type 2. Presented are data from the ACS cohort (*n* = 101) including patients suffering MI type 1 (*n* = 52) and type 2 (*n* = 10). **Left:** proportions of monocyte subsets in peripheral blood of patients with an MI type 1 or type 2. **Right:** box-and-whiskers plot of the distribution of the specific monocyte subsets in type 1 and type 2 MI (whiskers represent the interquartile range).

Figure 1 shows the distribution of different monocyte subpopulations (classical, non-classical and intermediate) in T1MI and T2MI patients. Visually, there was a slight shift from classical to non-classical in T1MI compared with T2MI (classical median (IQR) 510 (402–687) vs. 758 (426–916); non-classical median (IQR) 48 (33–58) vs. 35 (31–44)); however, none of the differences in subpopulations reached a *p*-value below 0.05 (see also Supplementary Table 2 for detailed information on the absolute and relative counts of the specific monocyte populations).

### Monocyte Subpopulations in Acute Myocardial Injury

**Figure 2:**
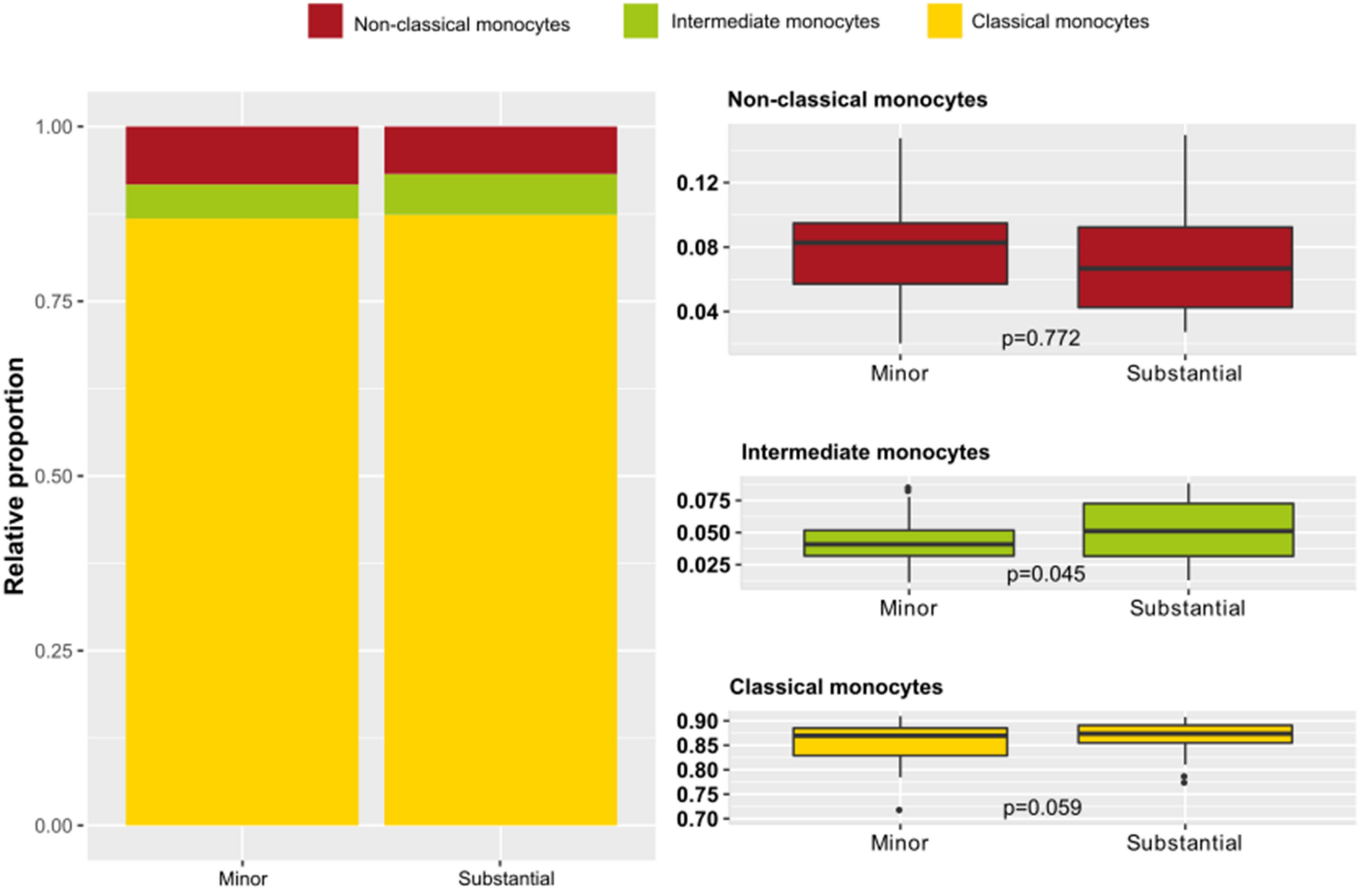
Monocyte subset proportions according to extent of myocardial injury in acute coronary syndrome (ACS) patients. Presented are data from the ACS cohort (*n* = 82) including patients with substantial (*n* = 42) and with minor myocardial injury (*n* = 40). ACS patients were stratified as having minor or substantial myocardial injury defined by high sensitivity cardiac troponin I values (99th percentile cut-off). **Left:** proportions of monocyte subsets in ACS patients with minor vs. substantial myocardial injury. **Right:** distribution of the specific monocyte subsets in relation to the extent of myocardial injury.

**Figure 3:**
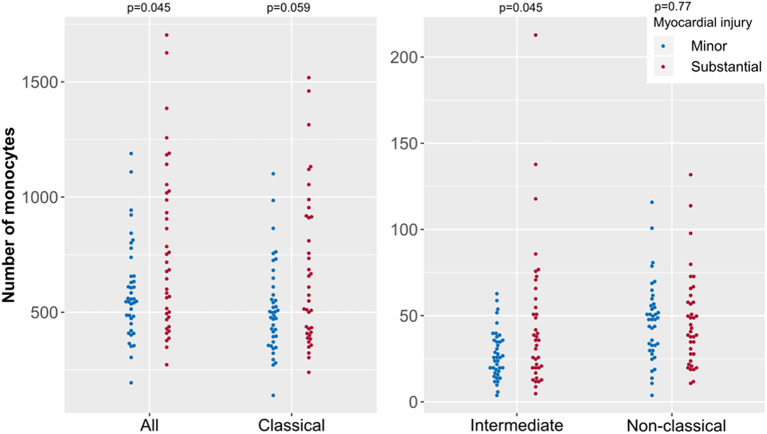
Absolute monocyte counts of specific subsets according to extent of myocardial injury in acute coronary syndrome (ACS). Presented are data from the ACS cohort (*n* = 82) including patients with substantial (*n* = 42) and with minor myocardial injury (*n* = 40). ACS patients were stratified as having minor or substantial myocardial injury defined by high sensitivity cardiac troponin I values (99th percentile cut-off). Each point represents the monocyte count of one patient.

A potential relationship between the extent of myocardial injury, irrespective of the final diagnosis, and the distribution of monocyte subsets was investigated (Figure 2). There was a higher proportion of intermediate monocytes in patients with substantial myocardial injury compared to patients with minor injury (*p* = 0.045). Patients with substantial injury showed only a tendency to have a lower proportion of non-classical monocytes (*p* = 0.772). To investigate this apparent difference in detail, absolute monocyte counts were further evaluated (Figure 3). In general, the total monocyte count was higher in patients with substantial injury compared with those with minor injury (*p* = 0.045). Regarding the different monocyte subset counts, the most relevant difference with respect to the extent of myocardial injury was observed in intermediate monocytes (*p* = 0.045), a subset that only accounts for 4.9 or 5.8% of total monocytes in patients with minor or substantial injury, respectively. The absolute counts in classical monocytes differed slightly in relation to myocardial injury, with higher counts in patients with substantial injury (*p* = 0.059). A multivariate analysis did not show relevant independent predictors of classical monocytes (see also Supplementary Table 4).

We further used the maximum CK value during the individual ACS time course as marker for peak injury. Here we observed a weak correlation with intermediate monocytes (rho = −0.273, *p* = 0.01), whereas no correlation was seen with classical (rho = −0.172, *p* = 0.112) or non-classical (rho = −0.102, *p* = 0.347) monocytes.

### Monocyte Subpopulations in Chronic Myocardial Injury

The non-ACS cohort of patients with suspected CCS was investigated to determine whether there was an association of monocyte subsets with extent of myocardial injury in patients without MI. Baseline characteristics of the CCS cohort are provided in Table 2. In comparison with patients showing no myocardial injury, patients showing substantial myocardial injury in this cohort were older (72.7 vs. 63.4 years, *p* < 0.001), more often male (78.4 vs. 54.3%, *p* = 0.003), and had a higher percentage of known diabetes mellitus (25.7 vs. 9.0%, *p* = 0.008), previously known coronary artery disease (54.1 vs. 34.8%, *p* = 0.02) and poorer renal function (creatinine 0.79 vs. 0.94 mg/dl, *p* = 0.001).

**Table 2:** Baseline characteristics of the chronic coronary syndrome (CCS) cohort.

**Chronic coronary syndrome (CCS) cohort**	**Type and unit**	**Overall CCS cohort**	**Substantial myocardial injury**	**Minor myocardial injury**	***p*–value**
		***n* = 144**	***n* = 74**	***n* = 70**	
Age	Median (IQR), [years]	66.84 (59.79–75.31)	72.7 (63.61–77.6)	63.44 (54.22–70.9)	<0.001
Male sex	*n* (%)	96 (66.67)	58 (78.38)	38 (54.29)	0.003
**Cardiovascular risk factors**					
Arterial hypertension	*n* (%)	121 (87.05)	66 (92.96)	55 (80.88)	0.111
Hypercholesterolemia	*n* (%)	100 (72.46)	54 (76.06)	46 (68.66)	0.37
Diabetes mellitus	*n* (%)	25 (17.73)	19 (25.68)	6 (8.96)	0.008
Smoking	*n* (%)	30 (27.27)	13 (21.31)	17 (34.69)	0.412
**History**					
Known coronary artery disease	*n* (%)	64 (44.76)	40 (54.05)	24 (34.78)	0.02
**Laboratory analyses**					
eGRF	Median (IQR), [ml/min/1.73 m^2^]	87.33 ± 26.62	82.12 ± 28.51	92.62 ± 23.59	0.02
Creatinine	Median (IQR), [mg/dl]	0.85 (0.73–1.02)	0.94 (0.74–1.23)	0.79 (0.69–0.92)	0.001
ss-cTnI	Median (IQR), [pg/mL]	3.06 (1.28–5.34)	5.28 (3.77–12.35)	1.24 (0.92–1.83)	<0.001
Cholesterol	Median (IQR), [mg/dl]	196 (164–229)	195 (167–222)	197 (163.5–237)	0.595
C-reactive protein	Median (IQR), [mg/dl]	0.2 (0.1–0.4)	0.2 (0.1–0.4)	0.2 (0.1–0.32)	0.91

*Data are shown stratified according to the extent of myocardial injury defined by super sensitivity cardiac troponin I values*.

*Data are presented as percentage, mean, or median as appropriate. eGFR denotes estimated glomerular filtration rate, ss-cTnI denotes super sensitivity cardiac troponin I*.

**Figure 4:**
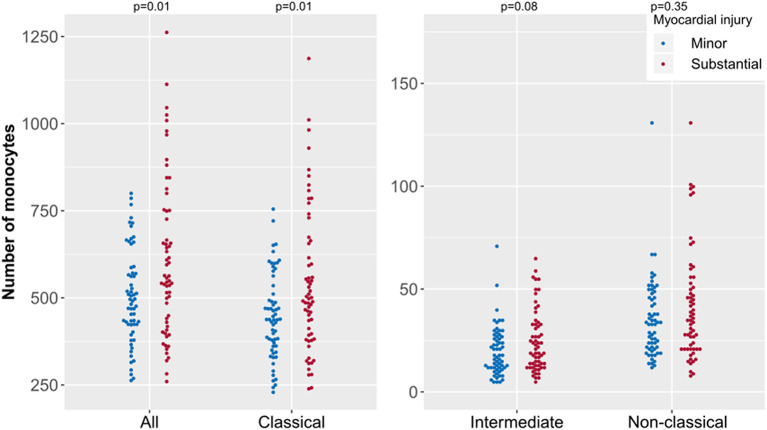
Absolute monocyte counts of specific subsets according to extent of myocardial injury in chronic coronary syndrome (CCS). Presented are data from the CCS cohort (*n* = 144) including patients with substantial (*n* = 74) and with minor myocardial injury (*n* = 70). CCS patients were stratified as having minor or substantial myocardial injury defined by super sensitivity cardiac troponin I values. Each point represents the monocyte count of one patient.

As in the ACS cohort, the total monocyte count was higher in patients of the CCS cohort presenting with substantial myocardial injury than in patients with minor injury (*p* = 0.01; Figure 4). Regarding the specific monocyte subsets in these non-acute patients, the presence of greater myocardial injury was associated with higher counts of classical monocytes (*p* = 0.01) and a tendency for higher counts of intermediate monocytes (*p* = 0.08).

The results of the groups with minor or substantial damage in the CCS cohort were analyzed by multivariate regression. The regression focused on the classical monocyte subpopulation: a 1% increase in troponin I caused a 0.062% increase in classical monocytes (*p* < 0.05). The other evaluated parameters (diabetes mellitus, known coronary artery disease, creatinine, eGFR, age, and sex) showed no relevant relationship to the amount of classical monocytes (Supplementary Table 5).

## Discussion

The inflammatory response component of MI is currently of great interest, as recent studies have documented that post-MI modulation of inflammation via medication reduces the occurrence of atherothrombotic events (14) and ischemic cardiovascular events (15). The early inflammatory response after MI is dominated by inflammatory cells such as monocytes, which are the initial protagonists of the complement cascade that migrate to the site of the event (2). However, the specific role of individual monocyte subsets in this context is still not fully understood.

This study presents data derived from two independent cohorts. In the first cohort of analyzed patients, that enrolled individuals with suspected ACS, a potential association of monocyte subsets with the type of MI and with the extent of myocardial damage was investigated. An association with the extent of myocardial damage was also evaluated in a second cohort comprising stable patients with suspected CCS having less myocardial injury.

The most important findings are: (i) type 1 as well as type 2 MI are associated with comparable proportions of the three monocyte subsets; (ii) monocyte subsets vary according to the extent of myocardial injury, irrespective of the presence of a diagnosed MI; (iii) even a small degree of myocardial injury, as observed in CCS patients, independently affects the distribution of monocyte subsets; (iv) this shift in monocyte subsets is mainly based on a change in intermediate and classical monocyte counts in ACS and CCS patients.

Given the difference in the pathophysiological background of T1MI and T2MI, one would expect a difference in monocyte subpopulations according to the type of MI. It has been shown that, in fact, inflammatory markers play a crucial role in the determination of the type of MI. For example, the type of MI can be differentiated with the help of a combination of the biomarkers MRP 8/14 and troponin I (11). Interestingly, in the present cohort no relevant relationship between the type of MI and monocyte subsets was observed. It must be emphasized that the diagnosis T1MI as well as the respective therapeutic regimen is clearly defined in the literature (16), whereas the group of patients diagnosed with T2MI is much more heterogeneous.

Previous research has shown that there is a difference in the monocyte subpopulations with respect to MI, in particular for patients with ST-elevation MI (STEMI) vs. non-STEMI (NSTEMI) (17). It is a common understanding that the electrocardiogram of STEMI patients is more often associated with a transmural infarction than that of NSTEMI patients. Hence, besides the pathophysiological differences, STEMI is associated with greater myocardial damage compared with NSTEMI. In the present study, cardiac troponins were used as indicators of the extent of damage to the heart due to their role as the current gold-standard biomarkers in MI (16) as well as their association with infarct size (18). Importantly, while previous study cohorts have often been limited to patients with a definite diagnosis of MI (4, 7, 17), our first analyzed patient cohort, named ACS cohort, comprised the whole spectrum of the working diagnoses of “suspected ACS” used in the emergency room, ranging from non-coronary origin of the clinical symptoms or unstable angina to a STEMI with total vessel occlusion.

In the ACS cohort, there was a shift in monocyte subsets according to the extent of myocardial injury, irrespective of the presence of an MI. Previous studies have also shown such a shift in monocyte subsets after a cardiac event, especially in the intermediate monocyte population (15, 19). Greater myocardial damage was associated with a higher monocyte count. This suggests that the observed shift in monocytes is at least in part dependent on the nature of the myocardial damage rather than on the specific cause of damage. To examine this idea further, monocyte subsets were evaluated in stable patients with suspected CCS. Even though the absolute troponin levels were markedly lower in the CCS cohort than in the ACS cohort, reflecting the smaller amount of myocardial injury overall, there was a clear shift in the monocyte subsets according to the degree of myocardial damage. The subset of intermediate monocytes in particular showed a relevant shift between patients with and without myocardial damage in both cohorts evaluated, suggesting an ACS-independent mechanism.

As expected, confounding factors such as age, sex, diabetes mellitus, previously known coronary artery disease, and renal function differed between patients with and without myocardial injury in the CCS cohort. However, multivariate regression analysis showed that the observed association of the amount of myocardial injury and the monocyte count was independent of these factors.

The predictive value of monocyte subsets regarding cardiovascular events occurring within 2 years after an invasively treated MI has been described, which underlines the relevance of changes in these subsets (20). Previous data suggest that changes in monocyte subsets are associated with pathophysiological aspects of ACS. For example, increased intermediate monocyte subsets were reported to be an independent predictor of thin-cap fibroatheroma (21), which itself is a precursor of plaque rupture (22) in ACS scenarios (23). The site of the culprit lesion may be a factor in promoting a shift in subsets (24). Differences in monocyte subsets have also been described between stable patients with coronary artery disease and controls (25). Further, monocyte subsets seem to be associated with prognosis in stable conditions, and it has been observed that the absolute counts of classical monocytes are predictive of major adverse cardiac events in patients with coronary artery disease. Intermediate monocytes express receptors that are crucial for angiogenesis and tissue repair (7). As such, they are predominantly involved in post-AMI healing process, whereas an enrichment of intermediate monocytes has been observed in the circulation of CCS patients and subjects with high risk plaques (26). This highlights the importance of CD14++ monocytes in cardiovascular diseases (27, 28). This upregulation of intermediate monocytes in patients with ACS and CCS might at least partly reflect accelerated mobilization from bone marrow (29). To fully understand the complex role of monocyte subsets in pathophysiological mechanisms further studies need to be performed and additional characterization features have to be considered. Recently, NFAT activating protein with ITAM motif 1 (NFAM1), which is associated with monocyte recruitment, has been identified as novel mediator affecting pathobiological progression of coronary artery disease. NFAM1 was highly elevated in ACS and CCS patients. NFAM1 is significantly higher expressed in classical and intermediate monocytes compared to non-classical monocytes (30). This might be an important link to our results, where a change in classical and intermediate monocyte subsets cell count but not in the non-classical monocyte subpopulation was observed in both ACS and CCS.

### Limitations

The present analysis has several limitations. First, due to the small numbers of patients, data from the analysis of each individual monocyte subpopulation could not be related to the final specific diagnoses in the ACS cohort. Second, the transferability to other cohorts with a different ethnicity is limited, as only patients from central Europe were enrolled in the two cohorts. Third, due to limited data availability on information between symptom onset and blood withdrawal in the ACS cohort it was not possible to evaluate a potential influence of differences in the time from symptom onset to measurement of monocyte subsets with adequate validity.

### Conclusion

The shift in monocyte subset counts after an MI is related to the severity of myocardial injury rather than the specific type of MI leading to that injury. This association of changes in monocyte subsets with myocardial injury applies to both acute and chronic coronary disease.

## Data Availability Statement

The raw data supporting the conclusions of this article will be made available by the authors, without undue reservation.

## Ethics Statement

The studies involving human participants were reviewed and approved by Local Ethics Committee of the Justus-Liebig-University Giessen. The patients/participants provided their written informed consent to participate in this study.

## Author Contributions

NA, CLip, and TK contributed to conception and design of the study and wrote the first draft of the manuscript. TK organized the database and supervised the project. NS, NA, and CLip performed data acquisition. NS, NA, CLip, DG, SV, and TK performed data analysis. NA and DG performed the statistical analysis. RK, BJ, JW, SK, OD, HN, CLie, and CH supported conduction of clinical study. All authors contributed to the interpretation of the results and contributed manuscript revision, read, and approved the submitted version.

## Funding

The present analysis is based on two cohorts that are part of the Kerckhoff Biomarker Registry (BioReg) that was financially supported by the Kerckhoff Heart Research Institute (KHFI) and the German Center for Cardiovascular Research (DZHK). The sponsors had no influence on the study design, statistical analyses, or draft of the paper.

## Conflict of Interest

The authors declare that the research was conducted in the absence of any commercial or financial relationships that could be construed as a potential conflict of interest.

## Publisher's Note

All claims expressed in this article are solely those of the authors and do not necessarily represent those of their affiliated organizations, or those of the publisher, the editors and the reviewers. Any product that may be evaluated in this article, or claim that may be made by its manufacturer, is not guaranteed or endorsed by the publisher.
