# Supplementary material for: Circulating Monocyte Subsets Are Associated With Extent of Myocardial Injury but Not With Type of Myocardial Infarction
Source: Front Cardiovasc Med. 2021 Nov 2;8:741890. doi: 10.3389/fcvm.2021.741890 (PMC8592918; doi:10.3389/fcvm.2021.741890)
Supplement: Supplementary file 1 [file Data_Sheet_1.PDF]

Supplementary Material

Supplementary Figures

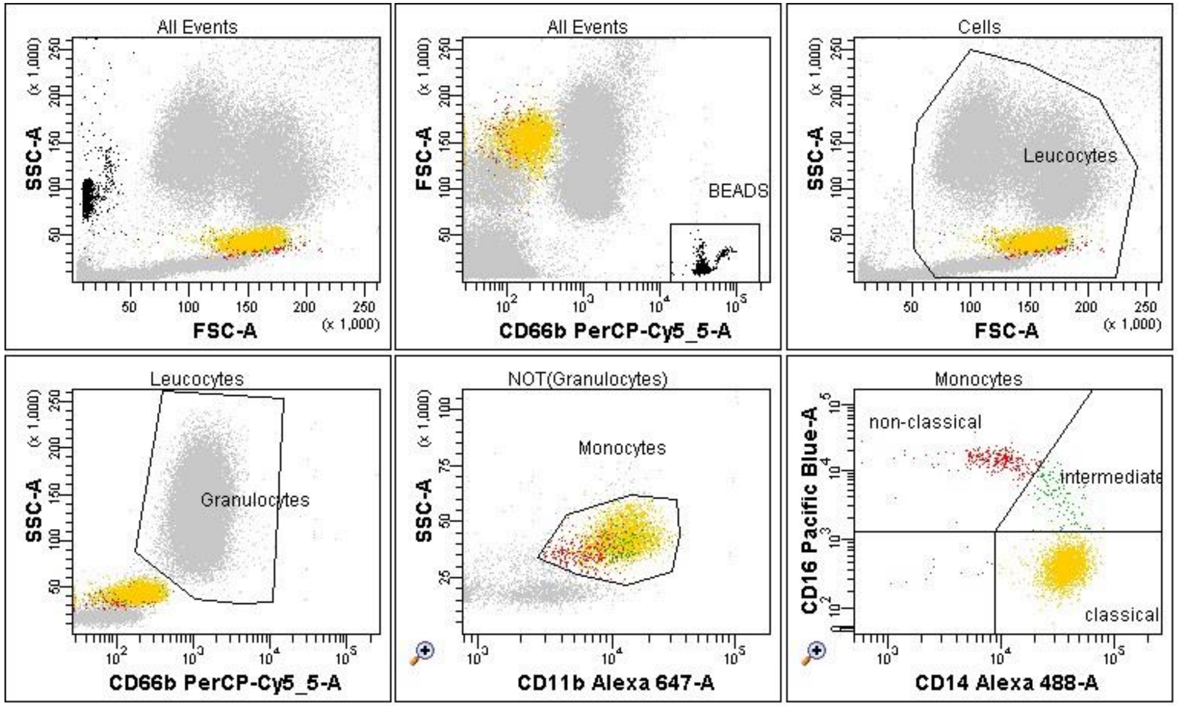

Supplementary Figure 1: Quantification of monocytes via flow cytometry

The figure shows an example for the gating of monocytes subsets in one individual patient of the ACS cohort. Black dots represent counting beads. Classical monocytes are shown in yellow, intermediate monocytes in green and non-classical monocytes in red.

Supplementary Tables

Acute Coronary Syndrome (ACS) cohort		Overall Cohort	Non-AMI	T1MI	T2MI			
	Type and Unit	n = 101	n = 39	n = 52	n = 10	Non-AMI vs. T1MI **p-value**	Non-AMI vs. T2MI **p-value**	T1MI vs. T2MI **p-value**
**Age**	median (IQR), [years]	69.18 (58.75-77.9)	67.46 (58.92-75.53)	70.29 (58.56-77.89)	71.82 (60.85-80.44)	0.572	0.412	0.547
**Male Gender**	n (%)	66 (65.35)	23 (58.97)	38 (73.08)	5 (50)	0.181	0.726	0.259
**Final diagnosis**								
**STEMI**	n (%)	5 (8.06)	0 (0)	5 (9.62)	0 (0)	0.068	1	0.582
**NSTEMI**	n (%)	57 (91.94)	0 (0)	47 (90.38)	10 (100)	*<0.001*	*<0.001*	0.582
**Cardiovascular Risk Factors**								
**Arterial Hypertension**	n (%)	61 (79.22)	26 (74.29)	29 (80.56)	6 (100)	0.387	0.721	1
**Hypercholesterolemia**	n (%)	43 (57.33)	18 (52.94)	21 (60)	4 (66.67)	0.67	1	1
**Diabetes Mellitus**	n (%)	24 (30.77)	12 (34.29)	10 (27.03)	2 (33.33)	0.225	0.702	1
**Smoking**	n (%)	29 (49.15)	12 (48)	15 (51.72)	2 (40)	1	0.702	0.713
**Family History**	n (%)	15 (27.78)	7 (29.17)	7 (26.92)	1 (25)	0.572	1	1
**History**								
**Known coronary artery disease**	n (%)	32 (50.79)	15 (48.39)	14 (53.85)	3 (50)	0.264	0.726	1
**Known atrial fibrillation**	n (%)	11 (14.67)	6 (17.65)	4 (11.43)	1 (16.67)	0.316	1	1
**Laboratory analyses**								
**eGFR**	median (IQR), [ml/min/1.73m^2]	85.53 (56.14-102.94)	88.78 (71.13-107.54)	85 (54.59-102.14)	75.72 (36.8-97.33)	0.373	0.128	0.255
**Creatinine**	median (IQR), [mg/dl]	0.89 (0.75-1.11)	0.82 (0.74-1.06)	0.91 (0.75-1.15)	0.94 (0.77-1.37)	0.231	0.316	0.632
**hs-cTnI**	median (IQR), [pg/mL]	28.2 (4-531.35)	3.85 (2.25-7.55)	490.8 (114.7-2918.5)	34.3 (8.85-43.2)	*<0.001*	0.057	*0.008*
**Cholesterol**	mean ± SD, [mg/dl]	196.88 ± 46.06	201.14 ± 49.77	198.18 ± 47.7	171 ± 16.97	0.896	0.226	0.188
**C-reactive protein**	median (IQR), [mg/dl]	0.3 (0.2-1.3)	0.3 (0.2-1.6)	0.3 (0.12-1.17)	0.5 (0.1-0.6)	0.48	0.967	0.702

Supplementary Table 1: Baseline characteristics of the *Acute Coronary Syndrome (ACS) cohort*

Data are shown stratified according to the final diagnosis acute myocardial infarction (AMI with non-ST and ST elevation MI; NSTEMI, STEMI) as well as according to the type of myocardial infarction including type 1 and type 2 myocardial infarction (T1MI and T2MI). Data are presented as percentage, mean, or median as appropriate. eGFR denotes estimated glomerular filtration rate, hs-cTnI denote high sensitivity cardiac troponin I.

Acute Coronary Syndrome (ACS) cohort		Overall Cohort	Non-AMI	T1MI	T2MI			
	**Type and Unit**	**n = 101**	**n = 39**	**n = 52**	**n = 10**	Non-AMI vs. T1MI **p-value**	Non-AMI vs. T2MI **p-value**	T1MI vs. T2MI **p-value**
**Monocytes, absolute count**								
**total monocytes**	median (IQR), [abs]	582 (470-815)	548 (437-760)	586 (472-787)	865 (475-1028)	0.369	0.251	0.485
**non-classical monocytes**	median (IQR), [abs]	43 (30-57)	43 (28-55)	48 (33-58)	35 (31-44)	0.38	0.741	0.283
**intermediate monocytes**	median (IQR), [abs]	27 (18-42)	26 (18.5-37)	27 (18-49)	31 (24-71)	0.599	0.256	0.384
**classical monocytes**	median (IQR), [abs]	505 (390-733)	487 (365.5-667)	510 (402-687)	758 (426-916)	0.478	0.214	0.414
**Monocytes, relative count**								
**non-classical monocytes part of total monocytes**	median (IQR), [abs]	0.07 (0.05-0.1)	0.07 (0.05-0.09)	0.08 (0.05-0.1)	0.05 (0.04-0.07)	0.764	0.114	0.153
**intermediate monocytes part of total monocytes**	median (IQR), [abs]	0.04 (0.03-0.07)	0.04 (0.03-0.06)	0.04 (0.03-0.07)	0.06 (0.03-0.07)	0.923	0.499	0.673
**classical monocytes part of total monocytes**	median (IQR), [abs]	0.88 (0.85-0.91)	0.88 (0.86-0.91)	0.87 (0.84-0.91)	0.89 (0.89-0.9)	0.622	0.585	0.308

Supplementary Table 2: Monocyte subsets in the *Acute Coronary Syndrome* (*ACS) cohort*

Data are shown stratified according to the final diagnosis acute myocardial infarction (AMI) including the type of myocardial infarction (type 1 and type 2 myocardial infarction; T1MI and T2MI). Data presented as median. eGFR denotes estimated glomerular filtration rate, hs-cTnI denote high sensitivity cardiac troponin I.

		Overall Cohort	Substantial Myocardial Injury	Minor Myocardial Injury	
**A** **Acute Coronary Syndrome**	**Type and Unit**	**n = 82**	**n = 42**	**n = 40**	**p-value**
**Monocytes, absolute count**					
**total monocytes**	median (IQR), [abs]	585.5 (476.5-812)	682.5 (484.75-1012.25)	554 (469.25-658.25)	*0.045*
**non-classical monocytes**	median (IQR), [abs]	46.5 (30-57.75)	42 (28-58)	48 (32.25-56.25)	0.772
**intermediate monocytes**	median (IQR), [abs]	27.5 (19.25-41.5)	36 (20-58.75)	26 (17.75-36)	*0.045*
**classical monocytes**	median (IQR), [abs]	510 (399-731.5)	563.5 (411.25-915)	493.5 (390.25-585.75)	0.059
**Monocytes, relative count**					
**non-classical monocytes part of total monocytes**	median (IQR), [abs]	0.07 (0.05-0.09)	0.06 (0.04-0.09)	0.08 (0.05-0.1)	0.073
**intermediate monocytes part of total monocytes**	median (IQR), [abs]	0.04 (0.03-0.07)	0.05 (0.03-0.07)	0.04 (0.03-0.06)	0.32
**classical monocytes part of total monocytes**	median (IQR), [abs]	0.88 (0.85-0.91)	0.88 (0.87-0.91)	0.88 (0.84-0.91)	0.616
**B** **Chronic Coronary Syndrome**		**n = 144**	**n = 74**	**n = 70**	**p-value**
**Monocytes, absolute count**					
**total monocytes**	median (IQR), [abs]	519 (425-658)	558 (431-750)	492 (425-572)	*0.014*
**non-classical monocytes**	median (IQR), [abs]	34 (21-48.75)	35 (21-51)	33 (22-46)	0.347
**intermediate monocytes**	median (IQR), [abs]	19 (12-28.75)	21 (13-33)	18 (12-27)	0.083
**classical monocytes**	median (IQR), [abs]	468.5 (380.25-583.25)	499 (394-657)	439 (363-512)	*0.012*
**Monocytes, relative count**					
**non-classical monocytes part of total monocytes**	median (IQR), [abs]	0.07 (0.05-0.09)	0.07 (0.05-0.09)	0.07 (0.05-0.09)	0.72
**intermediate monocytes part of total monocytes**	median (IQR), [abs]	0.04 (0.03-0.05)	0.03 (0.03-0.05)	0.04 (0.02-0.05)	0.9
**classical monocytes part of total monocytes**	median (IQR), [abs]	0.9 (0.86-0.92)	0.9 (0.86-0.92)	0.9 (0.87-0.92)	0.878

Supplementary Table 3: Influence of extent of myocardial injury on monocyte subsets in Acute Coronary Syndrome (**A**) and Chronic Coronary Syndrome (**B**) Data on absolute and relative counts of monocytes are shown stratified according to the extent of myocardial injury defined by increased troponin I levels. Data presented as median.

Acute Coronary Syndrome (ACS) cohort	Estimate	Std. Error	t-value	Pr(>|t|)
Log(**Classical Monocytes**)= Intercept	-80.40	61.40	-1.31	0.20
Log(**Troponin**)	0.01	0.02	0.56	0.58
Log(**Creatinine**)	19.10	13.70	1.40	0.17
Log(**eGFR**)	16.70	11.90	1.41	0.17
**Known artery disease**	0.00	0.00	-0.21	0.84
**Diabetes mellitus**	0.01	0.00	1.55	0.13
**Age**	0.06	0.04	1.55	0.13
**Gender**	4.90	3.54	1.38	0.17

Supplementary Table 4: Multivariate evaluation of the association of classical monocytes with myocardial injury.
Multivariate regression with classical monocytes as the intercept in the ACS cohort showing characteristics from *Table 1*. Not normally distributed variables were logarithmic (log)-transformed in the evaluation.

Chronic Coronary Syndrome (CCS) cohort	Estimate	Std. Error	t-value	Pr(>|t|)
Log(**Classical Monocytes**)= Intercept	-0.79	56.08	-0.01	0.989
Log(**Troponin**)	0.06	0.02	2.56	0.01 *
Log(**Creatinine**)	1.62	12.47	0.13	0.89
Log(**eGFR**)	1.31	10.81	0.12	0.90
**Known artery disease**	0.06	0.06	0.94	0.35
**Diabetes mellitus**	-0.09	0.09	-1.04	0.30
**Age**	0.01	0.03	0.16	0.88
**Gender**	0.38	3.23	0.12	0.91

Supplementary Table 5: Multivariate evaluation of the association of classical monocytes with myocardial injury.

Multivariate regression with classical monocytes as the intercept in the CCS cohort showing only the characteristics from *Table 2* with significant differences (p-value < 0.05) between Substantial Myocardial Injury and Minor Myocardial Injury. Not normally distributed variables were logarithmic (log)-transformed in the evaluation.
